# Mitochondrial Phylogenomics yields Strongly Supported Hypotheses for Ascaridomorph Nematodes

**DOI:** 10.1038/srep39248

**Published:** 2016-12-16

**Authors:** Guo-Hua Liu, Steven A. Nadler, Shan-Shan Liu, Magdalena Podolska, Stefano D’Amelio, Renfu Shao, Robin B. Gasser, Xing-Quan Zhu

**Affiliations:** 1 State Key Laboratory of Veterinary Etiological Biology, Key Laboratory of Veterinary Parasitology of Gansu Province, Lanzhou Veterinary Research Institute, Chinese Academy of Agricultural Sciences, Lanzhou, Gansu Province 730046, P. R. China; 2 College of Veterinary Medicine, Hunan Agricultural University, Changsha, Hunan Province 410128, P. R. China; 3 Department of Entomology and Nematology, University of California, Davis, CA 95616, USA; 4 National Marine Fisheries Research Institute, Kollataja 1, 81-332 Gdynia, Poland; 5 Department of Public Health and Infectious Diseases, Section of Parasitology, Sapienza University of Rome, Rome, Italy; 6 Genecology Research Centre, University of the Sunshine Coast, Queensland 4558, Australia; 7 Faculty of Veterinary and Agricultural Sciences, The University of Melbourne, Parkville, Victoria 3010, Australia

## Abstract

Ascaridomorph nematodes threaten the health of humans and other animals worldwide. Despite their medical, veterinary and economic importance, the identification of species lineages and establishing their phylogenetic relationships have proved difficult in some cases. Many working hypotheses regarding the phylogeny of ascaridomorphs have been based on single-locus data, most typically nuclear ribosomal RNA. Such single-locus hypotheses lack independent corroboration, and for nuclear rRNA typically lack resolution for deep relationships. As an alternative approach, we analyzed the mitochondrial (mt) genomes of anisakids (~14 kb) from different fish hosts in multiple countries, in combination with those of other ascaridomorphs available in the GenBank database. The circular mt genomes range from 13,948-14,019 bp in size and encode 12 protein-coding genes, 2 ribosomal RNAs and 22 transfer RNA genes. Our analysis showed that the *Pseudoterranova decipiens* complex consists of at least six cryptic species. In contrast, the hypothesis that *Contracaecum ogmorhini* represents a complex of cryptic species is not supported by mt genome data. Our analysis recovered several fundamental and uncontroversial ascaridomorph clades, including the monophyly of superfamilies and families, except for Ascaridiidae, which was consistent with the results based on nuclear rRNA analysis. In conclusion, mt genome analysis provided new insights into the phylogeny and taxonomy of ascaridomorph nematodes.

Parasitic nematodes (roundworms) cause diseases associated with serious morbidity and mortality in animals and substantial reductions in crop yields^1^. For example, species in the superfamily Ascaridoidea (ascaridoids)^2^, including species of *Anisakis, Ascaris, Baylisascaris, Contracaecum, Pseudoterranova* and *Toxocara*, are particularly important pathogens of various animals, and are transmissible to humans, where they cause diseases such as anisakidosis, ascariasis and toxocariasis^3^,^4^,^5^. In cases where ascaridoid larvae are directly responsible for human disease, infection is acquired through accidental ingestion of embryonated eggs (e.g., toxocariasis) or the consumption of larvae within raw meat or fish products (e.g., anisakidosis)^6^.

Although some species of ascaridoids can be readily identified and distinguished based on morphological features (e.g., *Toxocara cati, Toxocara canis* and *Toxascaris leonina*), molecular data have revealed cryptic species complexes within other morphological species (e.g., *Anisakis simplex, Contracaecum rudolphii* and *Pseudoterranova decipiens*). Some of these cryptic species show considerable differences in host and geographical ranges^7^,^8^,^9^. The biodiversity of ascaridoids is substantial, consistent with the broad range of hosts infected by these nematodes, including birds, mammals, reptiles, fish and amphibians^10^; there are also differences in ecology of particular host-parasite affiliations, and considerable genetic variation within species^11^. Important zoonotic disease complexes, such as anisakidosis, have substantial public health impact in several countries, including China, Germany, Japan, Netherlands and Spain^12^, emphasizing the need for improved diagnosis of the causative agents.

The accurate identification of ascaridoids to species is central to diagnosis, and also underpins fundamental and applied research of these important parasites. Challenges to diagnosis include the lack of species-level morphological features for most larvae, which are the stage responsible for disease in many cases. For many ascaridoid species complexes, identification to species is not possible based on morphology, such that nuclear or mitochondrial (mt) markers are necessary for identification. The first and second internal transcribed spacers of nuclear ribosomal DNA (ITS-1 and ITS-2 = ITS) have frequently been used as a species-specific marker in such cases^13^,^14^,^15^,^16^. By contrast, because of its relative conservation, the small subunit (SSU) of nuclear ribosomal RNA gene does not consistently provide sufficient sequence variation for the differentiation of ascaridoid species^17^. Most mt genes are much more variable in sequence than nuclear ribosomal RNA genes^18^,^19^, and recent studies have shown that amino acid sequences inferred from mt genome sequences are very useful for species identification as well as for investigating their inter-relationships^20^,^21^,^22^,^23^,^24^.

Detailed studies by Nadler *et al*.^25^,^26^, investigated the phylogenetic relationships of a range of Ascaridomorph taxa. Despite these advances, difficulties remain concerning the detection of cryptic species and the relationships among major lineages, such as families and superfamilies. For example, although some studies^27^,^28^ indicated that Anisakidae is more closely related to Ascarididae than to Toxocaridae, others^29^,^30^ have argued the opposite, and some have suggested that Anisakidae is sister to Toxocaridae + Ascarididae^31^,^33^. These results reflect inadequate resolution at higher levels for nuclear ribosomal data, conflicts between different inference methods, impacts of taxon sampling and limited phylogenetic signal from small data sets^34^,^35^. To overcome these limitations, we employed complete amino acid sequence data sets derived from mt genomes. Herein, we sequenced the complete mt genomes of six anisakids (Table 1), and reconstructed phylogenetic relationships using the data of these anisakids in combination with all mt genomic data sets available for ascaridomorph nematodes in public databases. Analyses of mt genome sequences in the present study have provided novel insights into the phylogenetic relationships of several major lineages of ascaridomorphs.

## Results

### Mitochondrial genomes of selected anisakids

**Figure 1:**
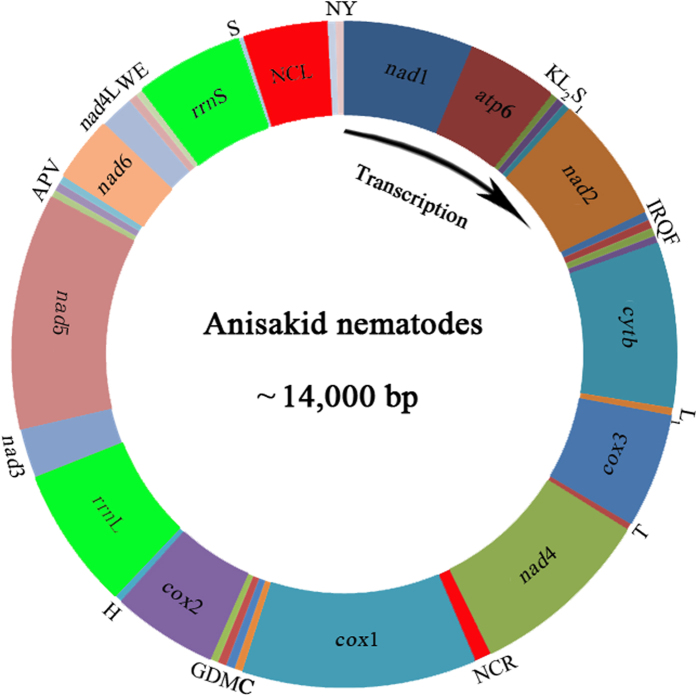
The mitochondrial genomes representing six anisakid nematodes. All genes are on the same DNA strand and are transcribed clockwise. Protein-coding and rRNA genes are indicated with the standard nomenclature. tRNA genes are indicated with the one-letter code of their corresponding amino acids. There are two tRNA genes for leucine: L_1_ for codons CUN and L_2_ for UUR; and two tRNA genes for serine: S_1_ for codons AGN and S_2_ for UCN. “NCL” refers to the large non-coding region. “NCR” refers to a small non-coding region.

The complete mt genomes of *Pseudoterranova krabbei, Pseudoterranova decipiens s.l.* (host *Chaenocephalus aceratus*) (PDCA), *Pseudoterranova decipiens s.l.* (host *Osmerus eperlanus*) (PDOE), *Pseudoterranova cattani, Pseudoterranova bulbosa* and three *Contracaecum ogmorhini s.l.* from different hosts [*Arctocephalus pusillus doriferus* (COAPD), *Arctocephalus pusillus pusillus* (COAPP) and *Zalophus californianus* (COZC)] were 13,948 bp, 13,965 bp, 13,962 bp, 13,950 bp, 13,957 bp, 14,019 bp, 14,013 bp and 14,010 bp, respectively. Each of the 8 mt genomes contains 36 genes, consisting of 12 protein-encoding, 2 rRNA, and 22 tRNA genes (Fig. 1), which is typical for chromadorean nematodes. Gene order (36 genes) for eight genomes is the same as published for *Anisakis simplex* (Anisakidae)^32^, *Ascaris* spp. (Ascarididae)^36^ *Toxocara* spp. (Toxocaridae)^31^, but distinct from *Ascaridia* spp. (Ascaridiidae)^20^ and *Cucullanus robustus* (Cucullanidae)^34^. In comparison to the *Ascaridia* species, at least three rearrangement events occurred in *An. simplex*. The 12 protein-coding genes have ATT, ATG or TTG as a start codon. Most of the protein-coding genes use TAA or TAG as a complete termination codon, except for *cox*1, *cox*2, *cox*3, *nad*5 and *nad*4L, which have abbreviated stop codons (TA or T). The tRNA genes range from 52 to 63 bp. Their predicted secondary structures (not shown) are similar to those of other anisakids^24^,^32^,^37^,^38^. The *rrn*L gene is located between tRNA-His and *nad*3, and *rrn*S gene is located between tRNA-Glu and tRNA-Ser^UCN^. The longer non-coding region (NCL) is located between the tRNA-Ser^UCN^ and tRNA-Asn, and the shorter non-coding region (NCR) is between *nad*4 and *cox*1.

### Comparative analyses of members of the *P. decipiens* complex and *C. ogmorhini*

Nucleotide sequences or the conceptually translated amino acid sequences from the protein-coding genes were aligned and compared to assess sequence divergence. Across the entire mt genome, the uncorrected nucleotide sequence divergence was 3.8–9.4% among members of the *P. decipiens* species complex. For the same group of species, the divergence across nucleotide and amino acid sequences for all protein-coding genes was 3.7–10.6% and 1.7–9.1%, respectively. A comparison of the nucleotide and amino acid divergence between *C. ogmorhini* from three different host species is provided in Table 2. Across the entire mt genome, the sequence divergence between COAPD and COAPP was 0.6% (93 nucleotide substitutions), 1.7% (244 nucleotide substitutions) between COAPP and COZC, and 1.7% (243 nucleotide substitutions) between COAPD and COZC. For the protein-coding genes, the respective divergences in nucleotide and amino acid sequences were 0.6% and 0.9% between COAPD and COAPP; 1.8% and 1.1% between COAPP and COZC; and 1.8% and 1.0% between COAPD and COZC, respectively.

### Phylogeny of ascaridoids inferred from mt genomic data sets

**Figure 2:**
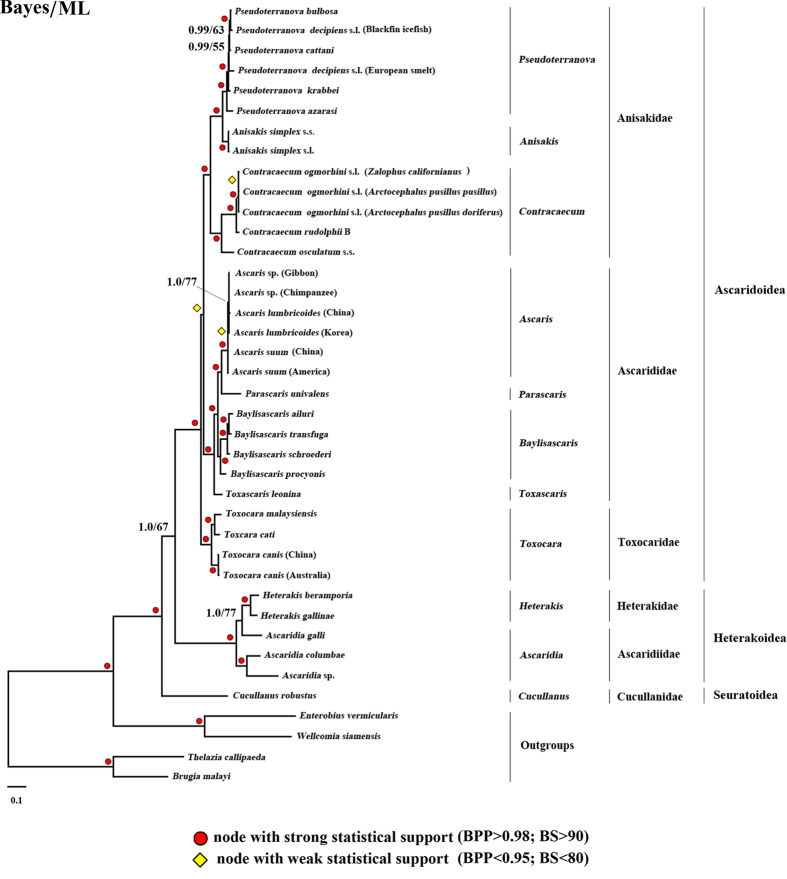
Phylogenetic relationships among ascaridoid nematodes inferred from Bayesian and maximum likelihood analyses of amino acid sequences for 12 mitochondrial genes. *Thelazia callipaeda, Brugia malayi, Wellcomia siamensis* and *Enterobius vermicularis* were used as outgroups. Bayesian posterior probability (BPP) and maximum likelihood bootstrap proportions (BS) are shown.

The 35 mt genomes analyzed include 20 species (29 taxa) representing Ascaridoidea, 4 species (5 taxa) of Heterakoidea and one species of Seuratoidea (Supplementary Table S1). Monophyly of the superfamilies Ascaridoidea (including three families Ascarididae, Toxocaridae and Anisakidae) and Heterakoidea (including two families Heterakidae and Ascaridiidae) were strongly supported in Bayesian (posterior probability, BPP = 1.0, Fig. 2) and maximum likelihood (ML) (bootstrap, BS = 100%, Fig. 2) analyses.

The Ascaridoidea, represented by families Ascarididae, Toxocaridae and Anisakidae, were monophyletic, with strong nodal support in Bayesian and ML analyses (BPP = 1.0, BS = 100%, Fig. 2). The family Ascarididae was monophyletic, with strong support in all analyses (BPP = 1.0, BS = 100%), as was the family Toxocaridae (BPP = 1.0, BS = 100%) and the family Anisakidae (BPP = 1.0, BS = 98%) (Fig. 2). Although there is strong support for monophyly of the Ascaridoidea (Anisakidae, Ascarididae, Toxocaridae), support for the sister-group relationship (Toxocaridae, (Anisakidae, Ascarididae)) differed greatly between the Bayesian and ML methods (BPP = 0.97, BS = 55%). The superfamily Heterakoidea was represented by five species from the families Heterakidae and Ascaridiidae, and was strongly supported as monophyletic (BPP = 1.0, BS = 100%, Fig. 2). The two species representing the Heterakidae were monophyletic, with strong support (BPP = 1.0, BS = 100%) in both analyses, but the three representatives of the family Ascaridiidae (all species of *Ascaridia*) were paraphyletic. One species of *Ascaridia, A. galli*, was sister to the two *Heterakis* species, whereas the remaining two *Ascaridia* taxa grouped together. The close relationship of *A. galli* to the two *Heterakis* species was strongly supported by Bayesian inference, but weakly supported in the ML analysis. Eight of the 11 in-group genera analyzed has two or more representative species (Fig. 2); all of these genera, except *Ascaridia*, were monophyletic, with strong BPP or BS support.

Within the family Ascarididae, the genus *Toxascaris* (represented by *T. leonina*) was sister to *Baylisascaris* spp., *Parascaris univalens* and *Ascaris* spp. (Fig. 2, BPP = 1.0, BS = 100%), supporting the genus *Toxascaris* as a member of the Ascarididae. In addition, *P. univalens* was sister to the genus *Ascaris* (represented by *A. lumbricoides* and *A. suum*) (Fig. 2, BPP = 1.0, BS = 99%), whereas four species of the genus *Baylisascaris* were a sister group to *Ascaris* plus *Parascaris* (Fig. 2, BPP = 1.0, BS = 100%). The genus *Toxocara* (represented by three species) was sister to the seven genera representing the Ascarididae plus Anisakidae. Within the family Anisakidae, *Pseudoterranova* (five species) was strongly supported as being sister to the genus *Anisakis* (Fig. 2, BPP = 1.0, BS = 100%), and *Contracaecum* (three species) was sister to *Anisakis* plus *Pseudoterranova* (Fig. 2, BPP = 1.0, BS = 98%).

## Discussion

Ascaridomorph nematodes are diverse; the superfamily Ascaridoidea alone contains more than 50 genera, including parasites of mammals, birds, amphibians, reptiles and fishes. Most molecular phylogenetic studies have focused on ascaridoid genera in the families Ascarididae and Anisakidae, and have mainly been based on nuclear ribosomal RNA genes^25^,^26^,^27^,^28^. More recently, some phylogenetic studies of the Ascaridida have used mt genetic^7^,^39^ and complete mt genomic data sets^29^,^32^,^34^,^35^. Here, we sequenced the mt genomes of eight representatives of the family Anisakidae and conducted comprehensive phylogenetic analyses of these nematodes and all other ascaridomorphs for which mt genomic data were available in databases. The present study has reinforced insights into the phylogenetic relationships of several lineages of ascaridomorphs and delivers a rich source of genetic markers for systematic, population genetic and epidemiological studies of these nematodes. In addition, such markers might also be used to explore the host specificity and zoonotic potential of these worms.

Our results are consistent with the division of the sampled ascaridomorph nematodes into three superfamilies: Ascaridoidea, Heterakoidea and Seuratoidea. Each of these superfamilies was monophyletic in our analysis with strong support, regardless of the analytical method. Within the superfamily Ascaridoidea, monophyly of the families Ascarididae, Anisakidae and Toxocaridae are each strongly supported by Bayesian and ML analyses. The best Bayesian consensus tree depicts a sister group relationship between Anisakidae and Ascarididae; however, this relationship is not reliably supported by ML bootstrap resampling. In other analyses of mt genomes, phylogenetic trees support a closer relationship between Anisakidae and Toxocaridae^29^,^34^, but these studies involved single representatives of each family. Analysis of nuclear SSU rRNA sequence data supports a closer relationship between Ascarididae and Toxocaridae^26^. In other analyses of molecular data, Anisakidae is sister to Ascarididae plus Toxocaridae^31^,^32^,^33^. Given the conflicts between these different analyses and datasets, it seems that additional loci will be required to resolve the sister-group relationships among the Anisakidae, Toxocaridae and Ascarididae.

Within the Ascarididae and Anisakidae, the relationships among genera based on mt genome sequence data is the same as that from a combined analysis of nuclear rRNA gene and *cox*2 as well as morphological data sets^39^. Relationships of species groups within *Contracaecum* are also consistent with the results inferred from nuclear rRNA gene sequences^40^.

The present study included five species from the superfamily Heterakoidea, two from the family Heterakidae and three from the family Ascaridiidae. In a previous investigation of mt genomes, monophyly of Ascaridiidae was well supported based on phylogenetic analysis of *Ascaridia* species^20^; however, inclusion of *Heterakis* species yields a non-monophyletic Ascardiidae due to the sister-group relationship of *A. galli* to *Heterakis*. Support for this result was strong from Bayesian inference (BPP = 1.0), but weak from ML analysis (BS = 77%). Some previous studies using morphological data indicated that the families Heterakidae and Ascaridiidae (Heterakoidea) were most closely related to the infra-order Oxyuridomorpha^41^. However, studies based on SSU rRNA gene sequence data have yielded conflicting results. For example, approximately half of the analyses performed by Nadler *et al*.^26^, based on SSU rRNA, yield a sister group relationship between Oxyuridomorpha and Heterakoidea. Previous studies of mt genomic data sets including those of *Ascaridia*^32^, *Heterakis*^42^ and *Ascaridia*^20^ supported a close relationship between Ascaridiidae and Ascaridomorpha. The improved sampling of Heterakoidea analyzed herein also supports a clade consisting of Ascaridoidea and Heterakoidea, rather than Heterakoidea and Oxyuridomorpha. Consistent with previous studies^20^,^32^,^43^, the current study provides strong support that *Ascaridia galli* is sister to the genus *Heterakis*, and supports the hypothesis that families Heterakidae and Ascaridiidae are more closely related to the infra-order Ascaridomorpha.

There is considerable value in employing mt genome markers to explore genetic variation within the genera *Pseudoterranova* and *C. ogmorhini*, because phenotypic differentiation is unreliable^44^. The *P. decipiens* complex consists of at least five cryptic species (genetically but morphologically indistinguishable), namely *P. decipiens, P. azarasi, P. cattani, P. krabbei* and *P. bulbosa*^45^,^46^. From the present study, nucleotide and amino acid sequences representing the mt proteome show a range of divergences (3.7–10.6% and 1.7–9.1%, respectively), and phylogenetic analyses indicate that these *Pseudoterranova* samples represent six distinct lineages (cf. Fig. 2). The nucleotide sequence differentiation (3.8–9.4%) detected in mt genomes was consistent with previous findings of differentiation (0–6.8%) in the sequences of the nuclear ITS rDNA from five *P. decipiens* species complex members^47^. In addition, we characterized here the mt genomes of three representatives of *C. ogmorhini* from different origins and hosts, and found sequence differences of <1.8% in the genes representing the mt proteome, which is comparable with levels detected between *A. suum* specimens from China and USA (1.5%)^36^, and *T. canis* from China and Australia (2.3%)^31^,^48^ (cf. Fig. 2). Taken together, these findings suggest that *C. ogmorhini* specimens from different host species represent a single species rather than sibling species, which contradicts previous conclusions from studies using nuclear ITS rDNA showing limited nucleotide variation (0.2–0.7%)^49^.

## Methods

### Genomic DNA samples of anisakid nematodes

Genomic DNA samples representing five sibling species of the *Pseudoterranova decipiens* complex and three populations of *C. ogmorhini s.l.* are listed in Table 1. These DNA samples had been used in our two previous studies^47^,^49^, and these anisakids had been identified preliminarily to species based on host preference, morphological characters and predilection sites^50^. The identities of these DNA samples were re-confirmed by PCR amplification and sequencing of the region spanning ITS-1, 5.8 S rRNA gene and ITS-2^47^,^49^. In addition, phylogenetic analysis of ITS data supports clustering of the *Pseudoterranova* in a previous study^49^.

### PCR amplification of the mt genomes of Anisakid nematodes

Based on the published mt genome sequences of anisakids^23^,^32^,^38^, we designed specific primers for long PCR amplification (Supplementary Table S2). We amplified the entire mt genome of individual specimens of anisakid nematodes by long PCR in four or five overlapping fragments, respectively. The four overlapping long-PCR fragments for *P. krabbei, P. bulbosa, P. cattani* and *P. decipiens s.l.* (Blackfin icefish) were between *nad*1 and *nad*4 (~5.0 kb), between *nad*4 and *rrn*L (~4.5 kb), between *rrn*L and *rrn*S (~3.5 kb) and between *rrn*S and *nad*1 (~2.0 kb). The five overlapping long-PCR fragments for *P. decipiens* s.l. (European smelt) were: *nad*1 to *cyt*b (~3.0 kb), *cyt*b to *nad*4 (~2.5 kb), *nad*4 to *cox*1 (~1.5 kb), *cox*1 to *rrn*L (~3.0 kb), and *rrn*L to *nad*1 (~5.0 kb) (cf. Fig. 1). The five overlapping long-PCR fragments for *C. ogmorhini* from different hosts were: *nad*1 to *cox*3 (~4.0 kb), *cox*3 to *cox*1 (~3.0 kb), *cox*1 to *rrn*L (~3.0 kb), *rrn*L to *rrn*S (~4.0 kb) and *rrn*S to *nad*1 (~1.8 kb). Each long-PCR reaction was performed in 25 μl using 2 mM MgCl_2_, 4.0 μL 0.2 mM each of dNTPs, 2.5 μL 10× rTaq buffer, 0.25 μL 2.5 μM of each primer, 0.25 μL 1.25 U rTaq polymerase (Takara), and 20–50 ng of total genomic DNA. The PCR conditions were: 92 °C for 2 min (initial denaturation), then 92 °C for 10 sec (denaturation), 54–59 °C for 30 sec (annealing) and 60 °C for 4–5 min (extension) for five cycles, followed by 92 °C for 2 min, then 92 °C for 10 sec, 54–59 °C for 30 sec, and 66 °C for 4–5 min for 30 cycles, and a final extension at 68 °C for 10 min. PCR amplicons were column-purified and then sequenced using a primer-walking strategy^51^.

### Sequence assembly and mt genome annotation

Sequence reads of anisakid nematodes were assembled with the program ContigExpress of the Vector NTI software package v.6.0 (Invitrogen, Carlsbad, CA). The mt genome sequences of anisakid nematodes were aligned with those of other anisakid nematodes available from the GenBank database^23^,^32^,^38^ using Clustal X 1.83^52^ to infer gene boundaries. Sequences of protein-coding genes were translated into amino acid sequences using the invertebrate mt genetic code in MEGA 5.0^53^. Translation initiation and termination codons were identified by comparison with those of the anisakid nematodes reported previously^23^,^32^,^38^. The secondary structures of 22 tRNA genes were predicted using tRNAscan-SE^54^, with manual adjustment^55^. Tandem repeats in the non-coding regions were found using Tandem Repeat Finder program (http://tandem.bu.edu/trf/trf.html)^56^.

### Phylogenetic analyses

All mt genome sequences of the infra-order Ascaridomorpha, along with those of selected Chromadorea outgroups, were obtained from GenBank and combined for phylogenetic analysis; *Thelazia callipaeda, Brugia malayi, Wellcomia siamensis* and *Enterobius vermicularis* were used to root the trees. Amino acid sequences inferred from the nucleotide sequences of 12 mt protein-coding genes were aligned individually using MAFFT v.7.122^57^ and were then concatenated to form a single dataset; ambiguously aligned regions were excluded using Gblocks 0.91b^58^ using default parameters.

Phylogenetic analyses were conducted using Bayesian (BI) and maximum likelihood (ML) inference. Maximum likelihood analysis was performed using RAxML v.7.0.3^59^ and implemented using the CIPRES web portal^60^. For ML analysis, the JTT (genes 1–8; *cyt*b, *cox*1, cox3, *atp*6, *nad*2, *nad*3, *nad*4L and *nad*5), LG (genes 9–10; *cox*2 and *nad*1), and MtArt (genes 11–12; *nad*4 and *nad*6) models were used as selected by ProtTest 2.4^61^ based on the Akaike information criterion (AIC). ML analysis was partitioned by gene, and bootstrap resampling was performed using the rapid bootstrapping option with 1,000 iterations. Bayesian analysis was also implemented using CIPRES and MrBayes 3.2.6^62^, and four independent Markov chains were run for 100,000 metropolis-coupled MCMC generations, sampling trees every 100 generations. The first 250 trees represented burn-in, and the remaining trees were used to produce Bayesian consensus trees. The analysis was performed until the potential scale reduction factor approached 1 and the average standard deviation of split frequencies was <0.01. For Bayesian analysis, the dataset was partitioned by gene, and the amino acid (aa) model for each gene was estimated from a mixture of models with fixed rate matrices and gamma distributed rates. With this analysis, each model contributes to the results in proportion to its posterior probability (BPP). Maximum-likelihood bootstrap (BS) support of >70% was considered strong support^63^. Phylograms were drawn using FigTree v.1.31 (http://tree.bio.ed.ac.uk/software/figtree/).

## Additional Information

**How to cite this article**: Liu, G.-H. *et al*. Mitochondrial Phylogenomics yields Strongly Supported Hypotheses for Ascaridomorph Nematodes. *Sci. Rep.* **6**, 39248; doi: 10.1038/srep39248 (2016).

**Publisher's note:** Springer Nature remains neutral with regard to jurisdictional claims in published maps and institutional affiliations.

## Supplementary Material

Supplementary Information

## Figures and Tables

**Table 1:** Anisakid nematodes representing *Pseudoterranova* spp. and *Contracaecum ogmorhini* populations from different hosts, geographical origins and GenBank accession numbers.

Species/population	Host species	Geographical origin	Mitochondrial genome Accession no	ITS-2 Accession no
*Pseudoterranova bulbosa*	*Erignathus barbatus* (Bearded seal)	Newfoundland, Canada	KU558720	AJ413971
*Pseudoterranova cattani*	*Otaria byronia* (South American sea lion)	Concepcion, Chile	KU558721	AJ413983
*Pseudoterranova decipiens s.l*.	*Chaenocephalus aceratus* (Blackfin icefish)	South Shetland Islands, Antarctica	KU558722	AJ413980
*Pseudoterranova decipiens s.l*.	*Osmerus eperlanus* (European smelt)	Elbe estuary, Germany	KU558723	AJ413978
*Pseudoterranova krabbei*	*Halichoerus grypus* (Grey seal)	Froya Island, Norway	KU558724	AJ413966
*Contracaecum ogmorhini s.l*.	*Arctocephalus pusillus doriferus*	Australia	KU558725	AJ291473
*Contracaecum ogmorhini s.l*.	*Arctocephalus pusillus pusillus*	South Africa	KU558726	AJ291472
*Contracaecum ogmorhini s.l*.	*Zalophus californianus*	Pacific Canada	KU558727	AJ291471

**Table 2:** Nucleotide (nt) and/or predicted amino acid (aa) sequence differences in each mt gene among three *Contracaecum ogmorhini* samples upon pairwise comparison.

Gene/region	Nt sequence length		COZC	Nt difference (%)			Number of aa		COZC	aa difference (%)		
	COAPD	COAPP		COAPD/COAPP	COAPP/COZC	COAPP/COZC	COAPD	COAPP		COAPD/COAPP	COAPP/COZC	COAPP/COZC
*atp*6	600	600	600	0.3	1.5	1.9	199	199	199	1.0	1.0	0
*nad*1	873	873	873	0.6	0.9	0.8	290	290	290	0	0.3	0.3
*nad*2	846	846	846	0.7	2.4	2.2	281	281	281	1.4	1.8	1.8
*nad*3	336	336	336	0.3	0.9	1.2	111	111	111	0	1.8	1.8
*nad*4	1230	1230	1230	0.4	2.9	3.2	409	409	409	1.0	1.2	1.7
*nad*4L	234	234	234	0.4	0.9	0.4	77	77	77	0	1.3	1.3
*nad*5	1582	1579	1582	0.6	0.3	0.6	527	526	527	0.8	0.6	0.6
*nad*6	435	435	434	0.5	0.7	0.2	144	144	144	0	0	0
*cox*1	1576	1576	1576	1.2	2.4	2.2	525	525	525	1.0	1.2	0.6
*cox*2	696	696	696	0.4	2.8	2.7	231	231	231	1.3	1.8	1.3
*cox*3	766	766	766	0.4	2.7	2.5	152	152	152	0.8	1.6	1.6
*cyt*b	1107	1107	1107	1.0	2.1	2.0	368	368	368	1.1	1.1	0.5
*rrn*S	691	690	690	0.4	0.7	0.9	—	—	—	—	—	—
*rrn*L	963	962	962	0.6	2.1	1.9	—	—	—	—	—	—
22 tRNAs	1253	1243	1254	0.7	1.4	1.5	—	—	—	—	—	—

COAPD: *Contracaecum ogmorhini s.l. (Arctocephalus pusillus doriferus*).

COAPP: *Contracaecum ogmorhini s.l. (Arctocephalus pusillus pusillus*).

COZC: *Contracaecum ogmorhini s.l. (Zalophus californianus*).
