# Supplementary material for: Mitochondrial Phylogenomics yields Strongly Supported Hypotheses for Ascaridomorph Nematodes
Source: Sci Rep. 2016 Dec 16;6:39248. doi: 10.1038/srep39248 (PMC5159812; doi:10.1038/srep39248)
Supplement: Supplementary Information [file srep39248-s1.pdf]

Supplementary Info

Mitochondrial Phylogenomics yields Strongly Supported Hypotheses for Ascaridomorph Nematodes

Guo-Hua Liu[1,2], Steven A. Nadler[3], Shan-Shan Liu[1], Magdalena Podolska[4], Stefano D'Amelio[5], Renfu Shao[6], Robin B. Gasser[7,*] & Xing-Quan Zhu[1,2,*]

[1]State Key Laboratory of Veterinary Etiological Biology, Key Laboratory of Veterinary Parasitology of Gansu Province, Lanzhou Veterinary Research Institute, Chinese Academy of Agricultural Sciences, Lanzhou, Gansu Province 730046, P. R. China, [2]College of Veterinary Medicine, Hunan Agricultural University, Changsha, Hunan Province 410128, P. R. China, [3]Department of Entomology and Nematology, University of California, Davis, CA 95616, USA, [4]National Marine Fisheries Research Institute, Kollataja 1, 81-332 Gdynia, Poland, [5]Department of Public Health and Infectious Diseases, Section of Parasitology, Sapienza University of Rome, Rome, Italy, [6]Genecology Research Centre, University of the Sunshine Coast, Queensland 4558, Australia, [7]Faculty of Veterinary and Agricultural Sciences, The University of Melbourne, Parkville, Victoria 3010, Australia. Correspondence and requests for materials should be addressed to R.B.G (email: robinbg@unimelb.edu.au) or X.Q.Z (email: xingquanzhu1@hotmail.com)

Table S1. Selected mitochondrial genome sequences of ascaridoids used for phylogenetic analysis in the present study, including the outgroups★.

Species	Family	Length (bp)	GenBank accession numbers
Anisakis simplex s.l	Anisakidae	13,916	NC_007934
Anisakis simplex s.s.	Anisakidae	13,926	KC965056
Ascaridia columbae	Ascaridiidae	13,931	JX624729
Ascaridia gallinae	Ascaridiidae	13,977	JX624728
Ascaridia sp.	Ascaridiidae	13,862	JX624730
Ascaris lumbricoides Korea isolate	Ascarididae	14,281	NC_016198
Ascaris lumbricoides China isolate	Ascarididae	14,303	HQ704900
Ascaris suum USA isolate	Ascarididae	14,284	NC_001327
Ascaris suum China isolate	Ascarididae	14,311	HQ704901
Ascaris sp. Chimpanzee	Ascarididae	14,268	KC839986
Ascaris sp. Gibbon	Ascarididae	14,274	KC839987
Baylisascaris ailuri	Ascarididae	14,657	NC_015925
Baylisascaris procyonis	Ascarididae	14,781	NC_016200
Baylisascaris schroederi	Ascarididae	14,778	NC_015927
Baylisascaris transfuga	Ascarididae	14,898	NC_015924
Brugia malayi★	Onchocercidae	13,657	NC_004298
Contracaecum ogmorhini s.l. (COAPD)	Anisakidae	14,019	KU558725
Contracaecum ogmorhini s.l. (COAPP)	Anisakidae	14,013	KU558726
Contracaecum ogmorhini s.l. (COZC)	Anisakidae	14,010	KU558727
Contracaecum osculatum s.s.	Anisakidae	13,823	NC_024037
Contracaecum rudolphii B	Anisakidae	14,022	FJ905109
Cucullanus robustus	Cucullanidae	13,972	NC_016128
Enterobius vermicularis★	Oxyuridae	14,010	NC_011300
Heterakis beramporia	Heterakidae	14,012	KU529972
Heterakis gallinae	Heterakidae	13,973	KU529973
Parascaris univalens	Ascarididae	13,920	KM067271
Pseudoterranova azarasi	Anisakidae	13,954	KR052144

Pseudoterranova bulbosa	Anisakidae	13,957	KU558720
Pseudoterranova cattani	Anisakidae	13,950	KU558721
Pseudoterranova decipiens s.l. (PDCA)	Anisakidae	13,965	KU558722
Pseudoterranova decipiens s.l. (PDOE)	Anisakidae	13,962	KU558723
Pseudoterranova krabbei	Anisakidae	13,948	KU558724
Toxocara canis China isolate	Ascarididae	14,029	NC_010690
Toxocara canis Australia isolate	Toxocaridae	14,162	EU730761
Toxocara cati	Toxocaridae	14,029	NC_010773
Toxocara malaysiensis	Toxocaridae	14,266	NC_010527
Toxascaris leonina	Ascarididae	14,310	NC_023504
Thelazia callipaeda★	Thelaziidae	13,668	JX069968
Wellcomia siamensis★	Oxyuridae	14,128	GQ332427

Contracaecum ogmorhini s.l. **(***Arctocephalus pusillus doriferus***)**: *Contracaecum ogmorhini s.l.* (COAPD)

Contracaecum ogmorhini s.l. **(** *Arctocephalus pusillus pusillus***)**: *Contracaecum ogmorhini s.l.* (COAPP)

Contracaecum ogmorhini s.l. **(** *Zalophus californianus***)**: *Contracaecum ogmorhini s.l.* (COZC)

Pseudoterranova decipiens s.l. (*Chaenocephalus aceratus*): *Pseudoterranova decipiens s.l.*(PDCA)

Pseudoterranova decipiens s.l. (*Osmerus eperlanus*): *Pseudoterranova decipiens s.l.* (PDOE)

Table S2. Sequences of primers for amplifying mitochondrial DNA of *Pseudoterranova decipiens* complex species and *Contracaecum ogmorhini* s.l. samples.

Primer	Sequence (5’ to 3’)	Amplification sequences	Reference
For Pk			
K-1F	CTACTCTTGTTAGTGGTGCTGTCA	*nad*1 to *nad*4	This study
K1-R	AACAACATCCTAGCAGTAGTAGGG	*nad*1 to *nad*4	This study
K2-F	GCTTTTTGTTCTATGCCTTTTCTT	*nad*4 to *rrn*L	This study
K2-R	TAA AAAACAAGCCTTCTCCTCTCT	*nad*4 to *rrn*L	This study
K3-F	GTCGTAACCAGATTCTTATGGTAG	*rrn*L to *rrn*S	This study
K3-R	CCAGGTACTAATCTGGTTTGTTTA	*rrn*L to *rrn*S	This study
K4-F	GTTCCAGAATAATCGGCTAGACTT	*rrn*S to *nad*1	This study
K4-R	TAAGACACTCTTTGACTCCTAGCC	*rrn*S to *nad*1	This study
For Pdca			
CA1-F	CTAAGTATGGAATAGTGGGGGCTA	*nad*1 to *nad*4	This study
CA1-R	GCAAGAAATACACAGGAAACTTCA	*nad*1 to *nad*4	This study
CA2-F	CTCTATGCCTTTTCTTTTTGTTTA	*nad*4 to *rrn*L	This study
CA2-R	ACAGTCTTAAAAAACAAGCCTTCT	*nad*4 to *rrn*L	This study
CA3-F	ATGGTAGTGATAAGTTTTACATCG	*rrn*L to *rrn*S	This study
CA3-R	TACTCAGCCTCCAAAGATAATTTA	*rrn*L to *rrn*S	This study
CA4-F	CCAGAATAATCGGCTAGACTTTAT	*rrn*S to *nad*1	This study
CA4-R	TTATAACCTCCAACAAGTTCTCTC	*rrn*S to *nad*1	This study
For Pc			
K-1F	CTACTCTTGTTAGTGGTGCTGTCA	*nad*1 to *nad*4	This study
K1-R	AACAACATCCTAGCAGTAGTAGGG	*nad*1 to *nad*4	This study
K2-F	GCTTTTTGTTCTATGCCTTTTCTT	*nad*4 to *rrn*L	This study
K2-R	TAA AAAACAAGCCTTCTCCTCTCT	*nad*4 to *rrn*L	This study
CA3-F	ATGGTAGTGATAAGTTTTACATCG	*rrn*L to *rrn*S	This study
CA3-R	TACTCAGCCTCCAAAGATAATTTA	*rrn*L to *rrn*S	This study
K4-F	GTTCCAGAATAATCGGCTAGACTT	*rrn*S to *nad*1	This study
K4-R	TAAGACACTCTTTGACTCCTAGCC	*rrn*S to *nad*1	This study
For Pdoe			
CF	TATTATACTAATGATGGTGCTTCT	*cytb*	This study
CR	AACATTGACCCAACCAACT	*cytb*	This study
N1-F	AGGGGAATATGGAGCTTTGTT	*nad*1 to *cytb*	This study
Cb1-R	AAAAACTCATCTGGGCTCATACTA	*nad*1 to *cytb*	This study
Cb-2F	AGTCATGTTAAGTTGGTTGG	*cytb* to *nad*4	This study
N4-2R	AAAGTCAAAATAAACCCTC	*cytb* to *nad*4	This study
N4-3F	TTGTTGGCTGGTTTGTTATTGA	*nad*4 to *cox*1	This study
C1-3R	ATTCTTAAAATAGCATACACCATCC	*nad*4 to *cox*1	This study
C1-4F	GGTTTGACGGGAGTTGTT	*cox*1 to *rrn*L	This study
Rl-4R	GCTACCTTAATGTCCTCACGCTA	*cox*1 to *rrn*L	This study
Rl-5F	CGGAGTTAACAGAAAATCATGTC	*rrn*L to *nad*1	This study
N1-5R	AGCACCTACTATTCCGTACTTAG	*rrn*L to *nad*1	This study

For Pb			
K1-F	CTACTCTTGTTAGTGGTGCTGTCA	*nad*1 to *nad*4	This study
K1-R	AACAACATCCTAGCAGTAGTAGGG	*nad*1 to *nad*4	This study
B2-F	GACTTTAACCTGTCTTGGG	*nad*4 to *rrn*L	This study
B2-R	CTACCTTAATGTCCTCAC	*nad*4 to *rrn*L	This study
B3-F	ATGAAATTGGCCCCTGCTC	*rrn*L to *rrn*S	This study
B3-R	TGATGGATGATTTGTACCG	*rrn*L to *rrn*S	This study
K4-F	GTTCCAGAATAATCGGCTAGACTT	*rrn*S to *nad*1	This study
K4-R	TAAGACACTCTTTGACTCCTAGCC	*rrn*S to *nad*1	This study
For *C. ogmorhini*			
CO1-F	GGTTTTAGCTGAGCTTAATCGTGC	*nad*1 to *cox*3	This study
CO1-R	GGTTCACCAGATGTATACCAAT	*nad* to *cox*3	This study
CO2-F	GTTATGGATGGTTTTAAG	*cox*3 to *cox*1	This study
CO2-R	ACTCTGACTAATAATACC	*cox*3 to *cox*1	This study
CO3-F	GGGCATCCTGAGGTTTATAT	*cox*1 to *rrn*L	This study
CO3-R	CTATCTCACGATGAATTAAAC	*cox*1 to *rrn*L	This study
CO4-F	CAATTGTTTTAGAGAGGAGGAGGC	*rrn*L to *rrn*S	This study
CO4-R	TACAACTTACTCCCCTATAGGC	*rrn*L to *rrn*S	This study
CO5-F	GGATAGTGCTGGCAGTTGGT	*rrn*S to *nad*1	This study
CO5-R	CTAATTCACTTTCACCCTCA	*rrn*S to *nad*1	This study
